# Confirmatory biopsy for the assessment of prostate cancer in men considering active surveillance: reference centre experience

**DOI:** 10.3332/ecancer.2016.633

**Published:** 2016-04-14

**Authors:** Cecilia Bosco, Gabriele Cozzi, Janette Kinsella, Roberto Bianchi, Peter Acher, Benjamin Challacombe, Rick Popert, Christian Brown, Gincy George, Mieke Van Hemelrijck, Declan Cahill

**Affiliations:** 1 King’s College London, Division of Cancer Studies, Cancer Epidemiology Group, London SE1 9RT, UK; 2 Guy’s and St Thomas’ Hospital NHS Foundation Trust, London SE1 9RT, UK; 3 King’s College Hospital NHS Foundation Trust, London SE5 9RS, UK; 4 Southend University Hospital NHS Foundation Trust, Southend SS0 0RY, UK; 5 European Institute of Urology, Division of Urology, Milan, Italy

**Keywords:** prostate cancer, active surveillance, confirmatory biopsy, transperineal sector biopsy, transrectal biopsy, prostate biopsy

## Abstract

**Objectives:**

To evaluate how accurate a 12-core transrectal biopsy derived low-risk prostate cancer diagnosis is for an active surveillance programme by comparing the histological outcome with that from confirmatory transperineal sector biopsy.

**Subjects and methods:**

The cohort included 166 men diagnosed with low volume Gleason score 3+3 prostate cancer on initial transrectal biopsy who also underwent a confirmatory biopsy. Both biopsy techniques were performed according to standard protocols and samples were taken for histopathology analysis. Subgroup analysis was performed according to disease severity at baseline to determine possible disease parameters of upgrading at confirmatory biopsy.

**Results:**

After confirmatory biopsy, 34% demonstrated Gleason score upgrade, out of which 25% were Gleason score 3+4 and 8.5% primary Gleason pattern 4. Results remained consistent for the subgroup analysis and a weak positive association, but not statistically significant, between prostate specific antigen (PSA), age, and percentage of positive cores, and PCa upgrading at confirmatory biopsy was found.

**Conclusion:**

In our single centre study, we found that one-third of patients had higher Gleason score at confirmatory biopsy. Furthermore 8.5% of these upgraders had a primary Gleason pattern 4. Our results together with previously published evidence highlight the need for the revision of current guidelines in prostate cancer diagnosis for the selection of men for active surveillance.

## Introduction

Active surveillance (AS) is a management option for men with low risk prostate cancer (PCa). The main advantage of AS is its low morbidity, but the existing nomograms based on one initial transrectal prostate biopsy alone have only modest accuracy in predicting the outcomes of these men managed by AS [1]. A recent systematic review estimated that men with PCa remain on AS between 2.2 and 5.4 years, with an estimated probability of discontinuation being 33% at five years and 55% at ten years. Discontinuation occurs because of either disease progression or patient’s anxiety [2]. Disease progression can potentially be avoided if high-risk disease is adequately assessed at the outset. Moreover, anxiety can be reduced by giving men with PCa more confidence about the low risk nature of their disease [3].

It is our standard practice to carry out a confirmative transperineal sector prostate biopsy (TPSB) on those men considering AS following an initial diagnosis of low risk PCa on transrectal ultra-sound (TRUS) biopsy. This protocol is based on our own experience and on the literature that shows about one-third of low risk PCa diagnosed on TRUS biopsy are likely to be misdiagnosed with actual disease at confirmatory biopsy [4, 5].

Therefore, the aim of this study was to evaluate how accurate a 12-core TRUS biopsy-derived low-risk PCa diagnosis is for AS by comparing the histological outcome with that from confirmatory biopsy.

## Patients and methods

This study is a retrospective review of data collected from 166 men consecutively diagnosed with PCa GS 3+3 between August 2007 and March 2012. Patients were selected on the basis of a 3+3 GS TRUS biopsy results for which they were placed under AS. Patients were selected from Guy’s hospital urology unit and data obtained from urology unit records.

**Figure 1.**
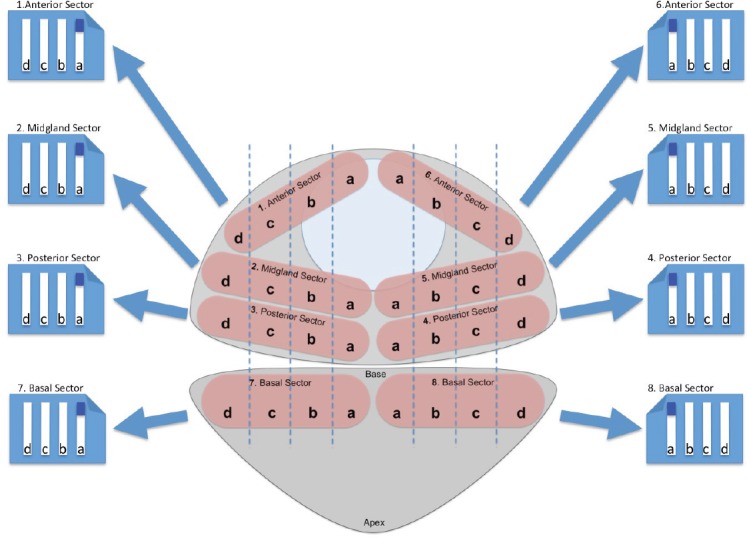
TPSB scheme used in our Institution. The biopsy starts from the right paraurethral region, sampling the anterior, mid, and posterior part of the gland. The ultrasound probe is then moved to show the most lateral part of the gland, which is again sampled in the anterior, mid and posterior regions. Next, the probe is moved medially and the regions of the gland comprised between the paraurethral and lateral region of the gland are sampled in their anterior, mid, and posterior part. The same procedure is performed on the left side.

TRUS biopsy was performed according to a standard protocol [6] under local anaesthesia and after antibiotic prophylaxis. A total of 12 cores were taken: two from the apex, and rest from lateral peripheral zone and the base at each side. All TPSB were carried out under general anaesthesia and with antibiotic prophylaxis. Men were placed in the lithotomy position with the scrotum taped to the abdomen to expose the perineum. Under transrectal ultrasound and brachytherapy template guidance, core biopsies were systematically taken on both sides of the prostate from the anterior, mid, posterior sectors [5] (Figure 1). Biopsies specimens were analysed in a single centre pathology laboratory including a quality control based upon interpathologist’s review.

Baseline characteristics between those whose GS upgraded between repeated biopsies and those whose GS did not upgrade were compared using ANOVA, t-tests, and chi-square test. An additional subgroup analysis was performed amongst those who presented with PSA ≥ 10 ng/mL and percentage of positive cores ≥20%. Predictors of disease progression (age, PSA, percentage of positive cores, and ethnicity) were evaluated using univariate and multivariate logistic regression analyses. Finally, a sensitivity analysis was performed limiting the time between biopsies to up to 15 months. The results did not vary from the analysis including up to 66 months. This analysis was performed to identify whether the variety in time between biopsies was having an effect on our overall findings. Given that the results from the sensitivity analysis did not differ from the entire cohort analysis, we showed the numbers of the entire cohort.

The statistical software used to analyse the data was Statplus 5.8.2.0.

Our study was approved as an audit by the local ethics and governance committee. All procedures were performed after informed consent was obtained.

## Results

The mean age of men in this cohort was 65.7 years (SD ±7 years). Median time between initial biopsy and confirmatory biopsy was six months (0.2–66 months). Mean age and PSA did not statistically differ between those whose GS upgraded between repeated biopsies and those whose GS remained the same (p-value: 0.06 and 0.21, respectively) (Table 1). Mean percentage of positive cores at initial biopsy did not show statistical differences (p = 0.64), however at confirmatory biopsy these means differed: 21.6 (±15.6) versus 7.1 (±9.4) for upgraders and no-upgraders respectively (p = <0.01) (Table 1).

After confirmatory biopsy, 56/166 men (34%) demonstrated GS upgrade, out of which 42/166 (25.5%) were upgraded to GS 3+4 and 14/166 (8.5%) to primary Gleason pattern 4 (11 to GS 4+3 and 3 to GS 4+5). When looking at those who presented PSA ≥ 10 ng/mL (n = 35) and percentage of positive cores ≥20 (n = 34) 48.6% and 44.1% were upgraded in each group respectively (Tables 1 and 2).

Logistic regression univariate and multivariate results showed a weak positive, but not statistically significant, association between predictors of disease progression (age, % positive cores, and PSA, odds ratio (OR) 1.04, 95% confidence interval (CI) 0.98–1.08, OR 1.68 95% CI 0.76–3.68, and OR 1.77 95% CI 0.83–3.78 respectively) and GS upgrading (Table 3).

## Discussion

We showed that in a cohort of men diagnosed with a GS 3+3 PCa at TRUS biopsy, confirmatory biopsy took place six months (median) after initial biopsy. Gleason score upgrade was detected in 33% of all men, with an upgrade to GS 3+4 in 25% of men, and an upgrade to primary Gleason pattern 4 or secondary Gleason pattern 5 in 9% of the cases. When looking at different subgroups of disease severity based on PSA values and percentage of positive cores on initial biopsy, the same pattern occurred: relative percentages showed that 45–50% of men upgraded to intermediate risk disease. Finally, a weak positive association was found between PSA ≥ 10 ng/mL, percentage of positive cores ≥20, age and ethnicity, and upgrading on logistic regression analysis.

Our results are in agreement with previous studies where the percentage of upgrade on confirmatory biopsies varies from 2.5% to 28% [4, 7, 8]. A possible reason for this difference in PCa detection could be attributed to the different techniques used for initial PCa diagnosis, i.e. using TRUS biopsy versus TPSB as a confirmatory biopsy. Since the description of the original sextant biopsy by Hodge *et al* [9], TRUS biopsy has been considered the gold standard approach for the diagnosis of PCa. However, the probability of missing a cancer with TRUS biopsy is thought to be >25%, even with saturation transrectal biopsy [10]. The low accuracy of TRUS biopsy has been previously analysed in several studies. Mitsuzuka *et al*. reported the outcomes of 87 men who met the PRIAS criteria, who underwent robot-assisted radical prostatectomy (RARP), following TRUS biopsy [11, 12]. Their results showed that 10% of men demonstrated pathological stage T3 on final pathology, 20% had GS upgrading to 4+3, and >69% had clinically significant cancer [11]. The radical prostatectomy outcomes of another large cohort of 919 PCa men who met a strict criteria for AS after TRUS biopsy (cT1c, PSA <10 ng/mL, a single positive biopsy, tumour length <3 mm, and Gleason score < 6) were similar to Mitsuzuka’s findings: GS was upgraded in 34% of men (1% presented GS 8–9), 11% of men had pT3 disease, and 1% had pT4, while 26% of men were found to have insignificant tumours [13].

Even though the transperineal approach shows higher detection rates, repeat TRUS biopsies have also been proven to reclassify PCa as clinically important. For instance in a study where both approaches were combined in the reassessment of AS candidates, 8% to 22% of men had prostate cancer reclassified as clinically important by repeat TRUS biopsy, while TPSB reclassified the disease in 41% to 85% of the men. Repeat TRUS biopsy results were assessed as not accurate in ruling out higher risk PCa [14]. However, given that TPSB is not widely available in every region/centre, this may not apply to every circumstance and TRUS biopsy should still be encouraged in those places where it is the only option available. TPSB is a technique that requires an operation room, an anesthesiologist, surgery assistants, and the trained professional (either urologist or nurse) that will be performing the biopsy. TRUS biopsy is performed under local anesthesia in the consulting room of an urologist/nurse. Furthermore, other studies have shown that using the same technique for both PCa diagnosis and stage confirmation, reclassification to a more severe grade of disease occurs in a significant number of cases [15–17].

Our results investigating potential predictors for the risk of upgrading are in agreement with the study by Motamedinia *et al* who also reported a positive association between PSA ≥ 10 ng/mL and disease upgrading at confirmatory biopsy [8].

Confidence in the diagnosis and stratification of men on AS is key in managing these men safely. Studies have shown that men with GS 3+4 at initial biopsy are more likely to be harbouring higher grade, high-risk disease [18–20]. Tosoian *et al* [21] showed that a better stratification for men with low risk and very low risk cancer PCa is important, since men with very low risk PCa had a significantly lower risk of adverse findings at surgery compared to those with low risk disease. These findings suggest that more precise techniques and protocols are needed to provide PCa men with confidence in AS and therefore a higher standard of care.

Regarding MRI-guided biopsies techniques, in recent publications where systematic biopsy techniques (either TPSB or TRUS) are compared with MRI fusion techniques, all authors observed that MRI fusion biopsies detected PCa of higher GS better than the systematic techniques. However, MRI techniques did miss clinical significant lower GS tumours. Thus they concluded that both techniques complement each other in the detection of clinically relevant PCa and should be performed concomitantly when possible in first and repeat biopsy [22–24].

Recently Epstein *et al* [25], have published a new PCa grading system based on radical prostatectomy specimens and biopsies of patients receiving radiotherapy. It mainly aims to reduce the risk of biochemical recurrence (BRC). TPSB or TRUS in AS are techniques used to establish the disease stage before radical treatment (curative radical prostatectmy (RP) or radiation therapy (RT)). Confirmatory TPSB or if not available, TRUS could be used to better apply this new grade group by assuring those that present at initial and at confirmatory biopsies GS of 3+3 or 3+4 (grade 1 and 2 of the new scoring system respectively) that AS is a safe treatment option, where no signs of disease progression are evident and has reduced comorbidities associated with curative treatments.

A major strength of this paper is the well-described and detailed cohort of patients with clearly defined suitability for AS. One limitation was the lack of detailed information on complications post confirmatory biopsy. We have, however, previously studied this in a cohort of 634 men and demonstrated that following TPSB the prevalence of urinary retention was very low (1.7%) with no cases of urosepsis being reported [5]. Data on PSA density, prostate volume, clinical stage, and cancer volume were not available from the patients records constituting the main limitation of this paper.

## Conclusion

In this single centre study, AS selection, defined by initial TRUS biopsy, we showed that 34% of men with apparent low risk disease were found to have higher-risk disease according to the results from systematic confirmatory biopsy. The accurate selection of low-risk disease is critical in order to inform the risk for progression for a successful AS programme. The higher accuracy of disease characterisation could thus increase the uptake of AS by improving the men’ and surgeons’ confidence by making a clear distinction between low, intermediate, and high-risk PCa. Evidence shows that the transperineal approach is more accurate in detecting higher GS disease at confirmatory biopsy, however as mentioned before the transrectal approach should be encouraged in those cases where it is the only approach available.

## Conflict of interest

None of the authors declare a conflict of interest.

## Figures and Tables

**Table 1.** Baseline characteristics of men with PCa in our cohort as well as histopathology results, by upgrade status.

	GS upgrade (n = 56 34%)	GS no-upgrade (n = 110 66%)	P-value
**Mean age (SD)**	67.1 (6.8)	64.9 (7.0)	0.06*
**Mean PSA (SD)**	8.85 (4.1)	7.7 (5.5)	0.21*
**% of positive cores at initial biopsy (%)**	15.1 (10.1)	14.3 (10.9)	0.64*
**% of positive cores at confirmatory biopsy (%)**	21.6 (15.6)	7.1 (9.4)	<0.01*
**Ethnicity**			
White	38 (67.8%)	77 (70%)	0.37**
Black	10 (17.8%)	24 (21.8%)	
Asian	2 (3.5%)	2 (1.81%)	
Mixed	0	3 (2.71%)	
Other/missing	5 (8.9%)	4 (3.63%)	
**Gleason score**			
3+3	0 (0%)	73 (66.3%)	<0.01**
3+4	42 (75.0%)	0 (0%)	
4+3	11 (19.6%)	0 (0%)	
4+5	3 (5.4%)	0 (0%)	
2+3	0 (0%)	1 (0.9%)	
Benign	0 (0%)	23 (20%)	
HGPIN	0 (0%)	13 (11.8%)	

* T-test.

** Chi-Square Test.

**Table 2.** Detailed overview of Gleason Score in subgroup of men with more severe PCa indicators at initial biopsy.

	PSA > 10 ng/mL n = 35 (21.1%)			>20% Positive Cores n = 34 (20.5%)		
**Gleason score**	**N of men**	**% Of entire cohort**	**% Of subgroup**	**N of men**	**% Of entire cohort**	**% Of subgroup**
**3+3 → 3+4:**	14	8.4%	40.0%	11	6.6%	32.4%
**3+3 → 4+3:**	1	0.6%	2.9%	4	2.4%	11.8%
**3+3 → 4+5:**	2	1.2%	5.7%	0	0.0%	0.0%
Total	17	10.2%	48.6%	15	9.0%	44.1%

**Table 3.** Odds ratios and 95% Confidence Intervals for risk of Gleason Score upgrade.

	Univariate			Multivariate		
	OR	P Value	95%CI	OR	P value	95%CI
**Age**	1.05	0.06	0.99–1.10	1.04	0.18	0.98–1.08
**Proportion of positive cores at initial biopsy**						
<20%	1.00	–	Ref	1.00	–	Ref
≥20%	1.75	0.15	0.81–3.78	1.68	0.20	0.76–3.68
**PSA**						
<10 ng/mL	1.00	–	Ref	1.00	–	Ref
≥10 ng/mL	2.05	0.05	0.99–4.23	1.77	0.14	0.83–3.78
**Ethnicity**						
White	1.00	–	Ref	–	–	–
Black	0.77	0.53	0.33–1.74	–	–	–
Asian	1.84	0.55	0.25–13.52	–	–	–

## References

[1] Wang SY (2013). Limited ability of existing nomograms to predict outcomes in men undergoing active surveillance for prostate cancer. BJU Int.

[2] Loeb S (2014). Overdiagnosis and overtreatment of prostate cancer. Eur Urol.

[3] Klotz L (2013). Active surveillance, quality of life, and cancer-related anxiety. Eur Urol.

[4] Ayres BE (2012). The role of transperineal template prostate biopsies in restaging men with prostate cancer managed by active surveillance. BJU Int.

[5] Vyas L (2014). Indications, results and safety profile of transperineal sector biopsies (TPSB) of the prostate: a single centre experience of 634 cases. BJU Int.

[6] Presti JC (2003). Extended peripheral zone biopsy schemes increase cancer detection rates and minimize variance in prostate specific antigen and age related cancer rates: results of a community multi-practice study. J Urol.

[7] Dall’Era MA (2012). Active surveillance for prostate cancer: a systematic review of the literature. Eur Urol.

[8] Motamedinia P (2012). Role of immediate confirmatory prostate biopsy to ensure accurate eligibility for active surveillance. Urol.

[9] Hodge KK, McNeal JE, Terris MK, Stamey TA (1989). Random systematic versus directed ultrasound guided transrectal core biopsies of the prostate. J Urol.

[10] Nafie S, Pal RP, Dormer JP, Khan MA (2014). Transperineal template prostate biopsies in men with raised PSA despite two previous sets of negative TRUS-guided prostate biopsies. World J Urol.

[11] Mitsuzuka K (2013). Pathological and biochemical outcomes after radical prostatectomy in men with low-risk prostate cancer meeting the Prostate Cancer International: Active surveillance criteria. BJU Int.

[12] van den Bergh RC (2007). Prospective validation of active surveillance in prostate cancer: the PRIAS study. Eur Urol.

[13] Beauval JB (2012). Pathologic findings in radical prostatectomy specimens from patients eligible for active surveillance with highly selective criteria: a multicenter study. Urol.

[14] Barzell WE (2012). Identifying candidates for active surveillance: an evaluation of the repeat biopsy strategy for men with favorable risk prostate cancer. J Urol.

[15] Berglund RK (2008). Pathological upgrading and up staging with immediate repeat biopsy in patients eligible for active surveillance. J Urol.

[16] ElShafei A (2015). Transrectal saturation biopsy improves risk stratification (reclassification) of patients with prostate cancer on active surveillance. Urol Practice.

[17] Venkitaraman R (2007). Predictors of histological disease progression in untreated, localized prostate cancer. J Urol.

[18] Crawford ED (2013). Clinical‐pathologic correlation between transperineal mapping biopsies of the prostate and three‐dimensional reconstruction of prostatectomy specimens. Prostate.

[19] El Hajj A (2013). Patient selection and pathological outcomes using currently available active surveillance criteria. BJU Int.

[20] Gershman B, Zietman AL, Feldman AS, McDougal WS (2013). Transperineal template-guided prostate biopsy for patients with persistently elevated PSA and multiple prior negative biopsies. Urol Oncol.

[21] Tosoian JJ, JohnBull E, Trock BJ, Landis P, Epstein JI, Partin AW, Walsh PC, Carter HB (2013). Pathological outcomes in men with low risk and very low risk prostate cancer: implications on the practice of active surveillance. J Urol.

[22] Borkowetz A, Platzek I, Toma M, Laniado M, Baretton G, Froehner M, Koch R, Wirth M, Zastrow S (2015). Comparison of systematic transrectal biopsy to transperineal magnetic resonance imaging/ultrasound-fusion biopsy for the diagnosis of prostate cancer. BJU Int.

[23] Radtke JP (2015). Comparative analysis of transperineal template saturation prostate biopsy versus magnetic resonance imaging targeted biopsy with magnetic resonance imaging-ultrasound fusion guidance. J Urol.

[24] Siddiqui MM (2013). Magnetic resonance imaging/ultrasound-fusion biopsy significantly upgrades prostate cancer versus systematic 12-core transrectal ultrasound biopsy. Eur Urol.

[25] Epstein JI (2016). A contemporary prostate cancer grading system: A validated alternative to the Gleason score. Eur Urol.

